# Public Health Perspectives of Preeclampsia in Developing Countries: Implication for Health System Strengthening

**DOI:** 10.1155/2011/481095

**Published:** 2011-04-04

**Authors:** Kayode O. Osungbade, Olusimbo K. Ige

**Affiliations:** ^1^Department of Health Policy and Management, Faculty of Public Health, College of Medicine and University College Hospital, University of Ibadan, P.M.B. 5017 General Post Office, Ibadan, Nigeria; ^2^Department of Community Medicine, University College Hospital, P.M.B. 5116, Ibadan, Nigeria

## Abstract

*Objectives*. Review of public health perspectives of preeclampsia in developing countries and implications for health system strengthening. *Methods*. Literature from Pubmed (MEDLINE), AJOL, Google Scholar, and Cochrane database were reviewed. *Results*. The prevalence of preeclampsia in developing countries ranges from 1.8% to 16.7%. Many challenges exist in the prediction, prevention, and management of preeclampsia. Promising prophylactic measures like low-dose aspirin and calcium supplementation need further evidence before recommendation for use in developing countries. Treatment remains prenatal care, timely diagnosis, proper management, and timely delivery. Prevailing household, community, and health system factors limiting effective control of preeclampsia in these countries were identified, and strategies to strengthen health systems were highlighted. *Conclusion*. Overcoming the prevailing challenges in the control of preeclampsia in developing countries hinges on the ability of health care systems to identify and manage women at high risk.

## 1. Introduction

Preeclampsia is a pregnancy-related hypertensive disorder occurring usually after 20 weeks of gestation. If left untreated, it progresses to eclampsia [1]. Preeclampsia and eclampsia are not distinct disorders but the manifestation of the spectrum of clinical symptoms of the same condition. The mildest disorder in this continuum is pregnancy-induced hypertension. In preeclampsia, hypertension and proteinuria are present, and when convulsions occur in addition to these signs, the condition is referred to as eclampsia [2]. 

Preeclampsia has remained a significant public health threat in both developed and developing countries contributing to maternal and perinatal morbidity and mortality globally [3–6]. However, the impact of the disease is felt more severely in developing countries [7, 8], where, unlike other more prevalent causes of maternal mortality (such as haemorrhage and sepsis), medical interventions may be ineffective due to late presentation of cases [9–11]. The problem is confounded by the continued mystery of the aetiology and the unpredictable nature of the disease [12]. Thus, the aim of this paper is to highlight the challenges militating against effective control of preeclampsia in developing countries and suggest measures which could be used to address them within the local context.

## 2. Methods

Literature providing evidence on the diagnosis, prevention, treatment, and overcoming challenges to the control of preeclampsia published between 2000 and 2010 were reviewed. Search terms included “preeclampsia”, “eclampsia”, “developing countries”, and “control”. These literatures were accessed from Pubmed (MEDLINE), AJOL, Google Scholar, and Cochrane Database of Systematic Reviews. Searches were also supplemented with recommendations from outside experts and reviews of bibliographies of other relevant articles and systematic reviews.

## 3. Results

### 3.1. Burden of Preeclampsia

Worldwide, the incidence of preeclampsia ranges between 2% and 10% of pregnancies. The incidence of preeclampsia, the precursor to eclampsia, varies greatly worldwide. WHO estimates the incidence of preeclampsia to be seven times higher in developing countries (2.8% of live births) than in developed countries (0.4%) [13]. The incidence of eclampsia in the developed countries of North America and Europe is similar and estimated to be about 5–7 cases per 10,000 deliveries. On the other hand, incidence of eclampsia in developing nations varies widely, ranging from 1 case per 100 pregnancies to 1 case per 1700 pregnancies [2, 14]. Rates from African countries such as South Africa, Egypt, Tanzania, and Ethiopia vary from 1.8% to 7.1% [15–18]. In Nigeria, prevalence ranges between 2% to 16.7% [19–21].

### 3.2. Public Health Perspectives of Preeclampsia

#### 3.2.1. Challenges in Detection and Prevention of Preeclampsia

Prevention of any disease process requires the availability of methods for prediction of those at high risk for the disorder. Although numerous clinical and biochemical tests have been proposed for prediction or early detection of preeclampsia, most remain unrealistic for general use in most developing countries. At present, there is not a single reliable and cost-effective screening test for preeclampsia which can be recommended for use in most developing countries [22]. Although some studies on uterine artery Doppler studies and first-trimester maternal serum markers for early detection of preeclampsia have shown promise [23–25]. There is not enough evidence to suggest their routine use in clinical practice, more so in resource poor settings [26].

In terms of prophylaxis, aspirin therapy has been shown to be beneficial in decreasing the occurrence of preeclampsia in specific populations, for example, those with abnormal second trimester uterine Doppler flow [27–30]. However, to recommend its widespread use in all patients is not judicious or evidence based. In the same vein, even though the Cochrane review has stated some benefit in calcium supplementation, particularly for those at greatest risk and those with low baseline calcium intake [31, 32], the problem of selecting appropriate patients to be started on the therapy can be burdensome from a public health perspective. Similarly, findings of earlier studies which had indicated the benefits of vitamin supplementation [33–39] have been refuted by a recent study by the WHO particularly for vitamins C and E [40].

#### 3.2.2. Challenges in the Management of Preeclampsia

It is evident that to tackle preeclampsia effectively in any population, functional health systems are imperative and so is access to health care. However, in the vast majority of developing countries particularly in Africa, health care access is limited due to a number of factors resulting in three levels of delay.

**Delay in the Decision to Seek Care**

Delayed responses at the household level to obstetric emergencies often arise as a result of inadequate information on when to seek help and sometimes on where to seek help [41–43]. This is worsened by lack of decision-making power, poverty, and the rising cost of health care [44, 45]. The consideration of user fees and the resultant catastrophic expenditure often result in fatal delays in care seeking [46]. Some sociodemographic (e.g., level of education and marital status) and cultural underpinnings of maternal health-seeking behaviour have also been documented [47].

**Delay in Reaching the Health Facility**

Lack of access to quality care has been said to be the main obstacle to reducing maternal mortality in low-income countries [46]. These are due to the location, distance, and lack of transport to health facilities. In Nigeria, for example, up to 50% of rural women live more than 5 km from the nearest hospital, and many have no way to get to health facilities except by walking—even when in labour [48]. The inequitable distribution of health facilities which is in favour of urban communities is also contributory [21]. Furthermore, the referral delays arising from the trajectory of visits to other orthodox and alternative medical practitioners have been documented to account for 46.4% of all cases of eclampsia [49, 50].

**Delays in Health Service Provision**

Delays which arise in health facilities have also been shown to prevent women from receiving the care they need before, during, and after childbirth. For instance, in many countries where the health insurance scheme is still in the teething stage, getting care in emergencies may be impossible for the poor or insured [51]. The attitudes of health service providers and perceived poor quality of care are also identified barriers [4, 52]. This is made worse by the lack of trained personnel and lack of equipment and supplies [45]. For instance, even though the efficacy of magnesium sulphate has been documented by several researchers [21, 53], studies have shown that magnesium sulphate was not routinely administered [41, 54], and use is often limited to teaching hospitals [55]. Lack of availability of the drug and appropriate health personnel required for its administration as well as cost were the frequently raised obstacles [56].

#### 3.2.3. Health Policies

At macro- and microhealth system levels, there are deficient policy guidelines and implementation. This has been blamed on poor data for decision making [56]. For example, reliable statistics about women dying due to eclampsia are difficult to obtain because of the poor quality of vital statistics registration systems and hospital records in many developing countries [57]. In addition, a sizable number of deliveries take place at home, and thus, there are no records at all for these births or their sequelae [56]. In addition, health policy development is usually not evidence based. This is because policy makers are often poorly informed of, and insufficiently involved in the use of research in policy development [58].

### 3.3. Recommendations

#### 3.3.1. Risk Assessment and Clinical Management

The WHO focused antenatal care strategy recommends screening for preeclampsia during the third antenatal visit at 32 weeks [59]. In developing countries, strategies for risk assessment should still be based on obstetric and medical history and clinical examination of women. Pregnant women should be assessed at their first antenatal clinic for risk factors of preeclampsia such as young age, nulliparity, first pregnancy after age of 35 years, obesity prior to the current pregnancy, multiple gestation, prior history of preeclampsia, diabetes mellitus, and hypertension [1, 60–62]. It is, however, important to note that the presence of these factors is not a surety to developing preeclampsia. It has been shown that screening for preeclampsia using maternal history was accurate in only 45.3% of cases [63]. 

Routine screening for preeclampsia based on measurement of blood pressure among all pregnant women should be practised as recommended by the World Health Organization [64, 65]. Where resources are available it is best to measure blood pressure using a mercury sphygmomanometer [66]. Urinalysis for protein should also be routinely done at every antenatal visit for pregnant women in developing countries as a complement to routine blood pressure measurement. The diagnostic criteria for preeclampsia developed by the National Blood Pressure Education Program Working Group which are still traditionally used in clinical practice are systolic blood pressure of 140 mm Hg or higher or a diastolic blood pressure of 90 mm Hg or higher on at least two occasions at least 4–6 hours apart occurring after 20 weeks of gestation in a woman whose blood pressure had previously been normal. In addition to this, the presence of proteinuria with excretion of 0.3 g or more of protein in a 24-hour urine specimen or 1+ or greater on two random urine samples collected four or more hours apart [67, 68].

Once recognised, depending on the severity, options of care include continued foetal and maternal evaluation, antihypertensive therapy, and timely delivery (the only definitive cure). There is significant evidence which supports the use of magnesium sulphate to prevent seizures in women with severe preeclampsia and eclampsia [1, 67, 69]. Magnesium sulphate has been compared with diazepam [70], phenytoin [71], and lytic cocktail [72] in randomized trials and results revealed that it produced a greater reduction in the risk of maternal death and recurrence of convulsions than other agents [73]. 

Furthermore, a continuum of care must be ensured. For women to benefit from the existing cost-effective interventions, they must have antenatal care in pregnancy, skilled care during delivery and postnatal care [74]. While antenatal care and skilled delivery could prevent seizures, postnatal visits are important to assess the recovery of the woman and to discuss what might happen in the future.

#### 3.3.2. Society and Community Interventions

Social factors have been recognised as influencing up to 27% of maternal deaths [75]. So, raising awareness of the need for women to reach emergency care without delay if complications arise during delivery is particularly critical. This is to ensure quick and effective medical intervention and to increase the chance of therapeutic success [1, 44]. Since many women deliver alone or with a relative [48], community members must also be trained to recognize danger signs and develop plans for emergencies, including transport to hospitals or health centre. Prompt health seeking behaviour is essential because reduction of the risk of death becomes more difficult when complications have developed [44].

#### 3.3.3. Health System Strengthening

The majority of intrapartum maternal deaths have been shown to occur in poorly performing health systems [76]. The evidence provided by some developing countries who have shown remarkable reduction in maternal mortality [75, 77, 78] shows that maternal safety must be made a priority health issue by the government and health workers. An increased focus on quality and accountability is also needed to secure the trust of consumers [79]. Political commitment to mobilize necessary resources to the health sector to improve the quality of emergency obstetric service must be shown by ensuring the availability of trained personnel, drugs, and equipment at every level of care. Referral services to emergency obstetric care must be prompt and affordable to limit delays when skills or facilities are lacking [80]. Improvements in service delivery can be achieved through the use of case management protocols for obstetric emergencies at each level of care and by monitoring standards of practice. Magnesium sulphate should be part of every developing country's list of essential drugs and national protocols on magnesium sulphate as the preferred treatment for preeclampsia and eclampsia should be developed and/or reinforced. To improve maternal health, barriers to accessing health services must be identified and addressed at all levels, with intensified efforts at community mobilization and engagement [81]. The use of data to improve quality of care is also important especially improving reporting systems and record-keeping practices to estimate disease burden to aid service planning and delivery [78]. Maternal death audits would aid the understanding of the pathways to survival and death and help in local efforts at improvement. It would also aid in identifying substandard care and avoidable factors in eclampsia-related maternal deaths [56, 82].

## 4. Conclusions

With the target of the Millennium Development Goals in sight, preeclampsia/eclampsia needs to be identified as a priority area in reducing maternal mortality in developing countries. Since the mainstay of control remains health care based strategies, national governments and supporting agencies should channel efforts at strengthening the public health systems and improving access to trained health care providers. Further research is needed to understand the causes and the best preventive strategies for preeclampsia specific to geographic areas. However, based on current data, better access to appropriate obstetric care, particularly during labour and delivery and better screening and treatment of identified cases should reduce preeclampsia rates in developing countries.
